# Weekly Medical Review Visiting List

**Published:** 1891-01

**Authors:** 


WEEKLY MEDICAL REVIEW. Pocket reference book, and visiting list perpetual. St. Louis, Mo. J. H. Chambers & Co.

From the number of visiting lists now published, it is very hard indeed for the physician to make a selection, all have their individual attractions and merits, while in many respects like most of the others. The Weekly Review reference book and visiting list differs from some in having very serviceable chapters on the clinical and chemical examination of the urine and a diagnostic table of eruptive fevers, together with much other interesting and appropriate matter for reference. It is neatly bound, and of very suitable size for the pocket.

